# Transfemoral Osseointegration in Association With Total Hip Replacement: Observational Cohort Study of Patients With Follow-Up Exceeding 2 Years

**DOI:** 10.1016/j.artd.2024.101463

**Published:** 2024-07-20

**Authors:** Jason Shih Hoellwarth, Amanullah Haidary, Kevin Tetsworth, Atiya Oomatia, Munjed Al Muderis

**Affiliations:** a Limb Lengthening and Complex Reconstruction Service, Osseointegration Limb Replacement Center, Hospital for Special Surgery, New York, NY, USA; b Western Sydney University School of Medicine, Campbelltown, New South Wales, Australia; c Department of Orthopaedic Surgery, Royal Brisbane and Women's Hospital, Herston, Queensland, Australia; d Limb Reconstruction Centre, Macquarie University Hospital, Macquarie University, Macquarie Park, Australia

**Keywords:** Osseointegration, Total hip arthroplasty, Transfemoral amputation, Amputation, Amputee

## Abstract

**Background:**

Some amputees with transfemoral osseointegration (TFOI) have ipsilateral hip arthritis which can be addressed with total hip arthroplasty (THA). This study reported the medium-term outcomes of THA in association with TFOI (THA + TFOI).

**Methods:**

Retrospective review was performed for eight patients with THA + TFOI performed at least 2 years prior. Primary outcomes include complications prompting surgical intervention. Secondary outcomes include changes in mobility (K-level, 6-minute walk test [6MWT], timed up and go) and patient-reported measures (hip pain, daily prosthesis wear hours, Questionnaire for Persons with a Transfemoral Amputation, and Short Form 36 [SF36]).

**Results:**

One patient died after 11 months (cancer); he was included to maximally report complications but excluded from mobility and reported outcomes. Three patients required subsequent surgeries: Two had skin refashioning, and the other underwent hip debridement of the replaced joint with subsequent removal of the TFOI. No perioperative complications, fractures, or arthroplasty explantations occurred. All patients reported complete hip pain relief. Of 6 patients reporting prosthesis wear time, 2 (33%) wore their prosthetic leg at least 4 hours daily before, vs all (100%) who did afterward (*P* = .061). K-levels improved in all responding patients. All 5 wheelchair-bound patients achieved and maintained ambulation. The Questionnaire for Persons with a Transfemoral Amputation and Short Form 36 did not significantly change.

**Conclusions:**

THA + TFOI does not appear to pose an inevitable risk for prosthetic hip infection and may improve mobility and enhance quality of life (QOL) for transfemoral amputees with concurrent arthritic hip pain who are dissatisfied with their outcome following traditional socket prosthesis rehabilitation.

## Introduction

**Figure 1:**
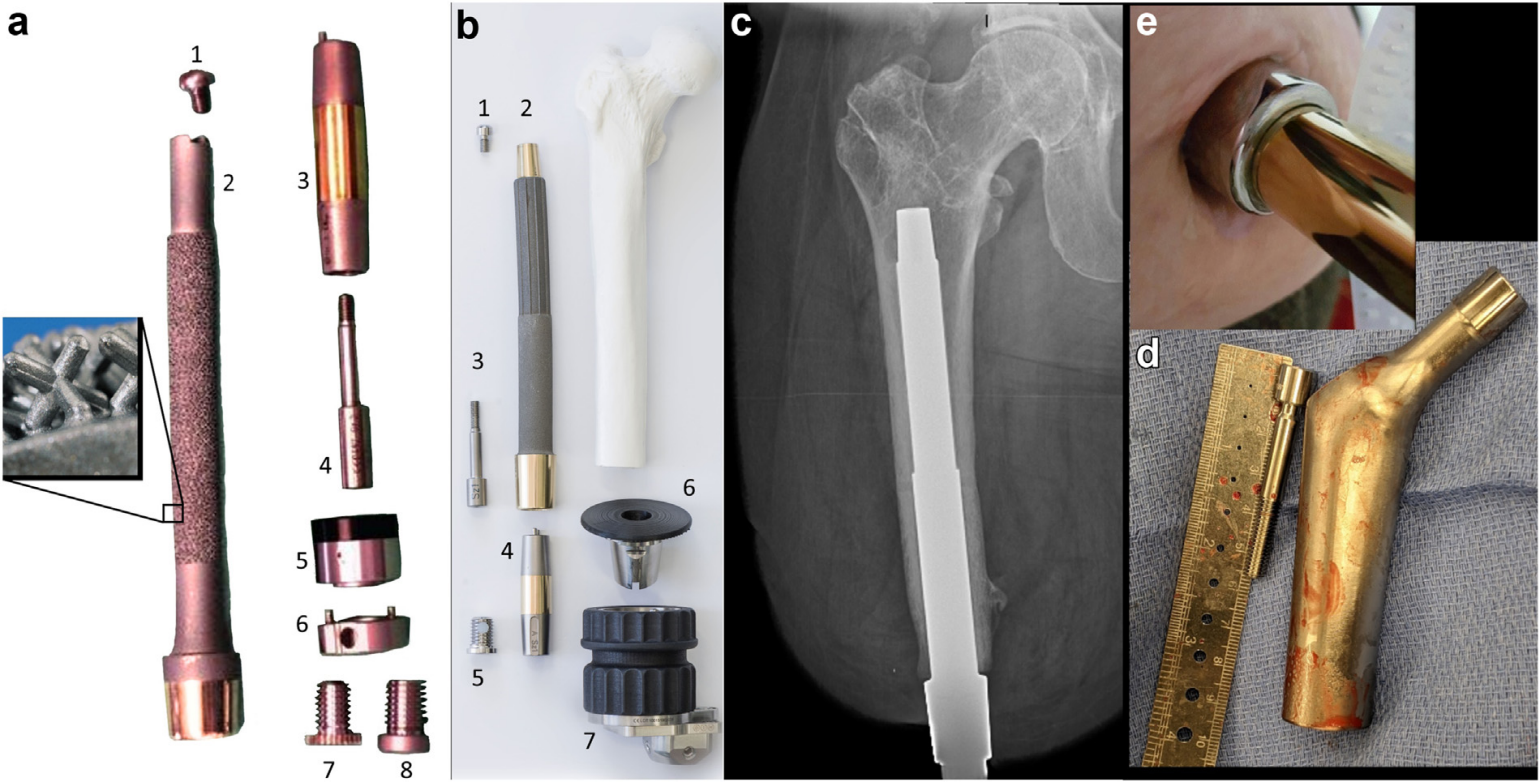
Depiction of osseointegration and total hip arthroplasty models. (a) Integrated limb prosthesis (ILP). Cobalt-chrome alloy with textured surface of 1.5-mm Czech hedgehogs (a 3-dimensional plus sign design, shown in zoom-in box). 1, proximal cap screw; 2, ILP body with main portion textured, distal flare untextured, abutment highly polished with titanium niobium oxynitride ceramic surface; 3, dual cone abutment adapter; 4, safety screw; 5, taper sleeve; 6, distal bushing; 7, permanent locking propeller screw; and 8, temporary cover screw. (Figure adapted, by permission, from Springer Nature: Springer Nature, Der Orthopäde. Juhnke DL, Aschoff HH. Endo-Exo-Prothesen nach Gliedmaßenamputation. Der Orthopäde. 2015 Jun; 44[6]:419-25. Epub 2015 May 14. Copyright 2015pringer Nature: Springer Nature, Operative Orthopädie und Traumatologie. Aschoff HH, Clausen A, Tsoumpris K, Hoffmeister T. Implantation der Endo-Exo-Femurprothese zur Verbesserung der Mobilität amputierter Patienten. Oper Orthop Traumatol. 2011 Dec;23[5]:462-72. German. And also Operative Orthopädie und Traumatologie. Aschoff HH, Clausen A, Tsoumpris K, Hoffmeister T. Implantation der Endo-Exo-Femurprothese zur Verbesserung der Mobilität amputierter Patienten. Oper Orthop Traumatol. 2011 Dec;23[5]:462-72. German.) (b) Osseointegrated prosthetic limb (OPL)-forged titanium alloy, stem-shaped implant whose surfaces have a plasma-sprayed coating, up to 0.5 mm thickness, to promote bone ingrowth and rapid integration. The external portions of the collars are treated with titanium niobium oxynitride ceramic to promote smooth soft-tissue gliding, limiting the probability of symptomatic soft-tissue adhesion and tethering. Proximal fluted fins provide initial rotational stability, akin to a Wagner-style hip arthroplasty stem. Exploded view, with the components arranged at approximately the proximal-distal levels in which they would be once assembled and implanted in a patient who had undergone a femoral amputation. 1, proximal cap screw; 2, OPL body; 3, safety screw; 4, dual cone abutment adapter; 5, permanent locking propeller screw; 6, proximal connector; and 7, prosthetic connector. (c) Radiograph of OPL Type A in a patient who had undergone a femoral amputation. (d) Example of a smooth surface total hip adapter which would be mated to a transfemoral OPL stem via the OPL’s proximal taper and screw. (e) Example of the skin portal where the patient’s internal implant abutment receives the external dual cone prosthesis connector.

Patients with transfemoral amputation face many challenges throughout their rehabilitation journey. Some of those challenges are directly related to interface issues with a traditional socket prosthesis (TSP), notably skin and fit issues [1,2]. Transcutaneous osseointegration, recently reviewed [3,4] (Fig. 1), has proven to be a safe, viable alternative to a TSP [5,6]. By replacing the socket with a skeletally linked interface, improved mobility and quality of life (QOL) is conferred for the vast majority of lower limb amputees [7,8].

An additional challenge to rehabilitation of patients with transfemoral amputation can result from degenerative hip joint disease, whether directly or indirectly related to the amputation etiology. Many people, including those with transfemoral amputation, may develop painful hip joint arthritis, which can severely limit mobility and diminish QOL. The combination of transfemoral osseointegration (TFOI) with total hip arthroplasty (THA) can address both these mobility and QOL issues for such patients. We previously reported on the first use of THA directly linked with TFOI (THA + TFOI) for 3 patients followed up to 2.5 years, establishing the viability of this surgical reconstruction [9]. No other article has described this reconstructive treatment combination.

The current study was performed to provide longer-term follow-up data regarding THA + TFOI, in order to improve the understanding of the risks and benefits. The primary aim was to evaluate the safety of THA + TFOI (adverse events and complications prompting additional surgery). The secondary aim was to describe the impact of THA + TFOI on these individuals' mobility and QOL.

## Material and methods

Institutional ethics approval was obtained for this study. This report follows the STrengthening the Reporting of OBservational studies in Epidemiology (STROBE) reporting guidelines for observational studies.

### Study design

#### Participants/selection

Our prospectively maintained osseointegration registry was retrospectively reviewed for the following study inclusion criteria: all patients who had TFOI and who also had ipsilateral THA (simultaneously or metachronous), with at least 2 years of follow-up or who died during that period. This identified 8 patients whose surgeries were performed between December 2013 and August 2020.

Patients indicated for osseointegration are generally skeletally mature adults who (1) report dissatisfaction with their TSP due to pain or limited mobility; (2) have an intact limb with incapacitating pain, complex deformity, or profound distal weakness whose functional capacity would likely be improved by amputation; or (3) have recently undergone amputation and who prefer osseointegration to TSP. Contraindications to osseointegration include modifiable compromises to successful skin and/or bone healing, such as active infection or malignancy, although upon treatment of the modifiable compromises, most patients may later be considered suitable. The indications for THA include severe hip joint pain and/or functional limitations secondary to cartilage degeneration. There were no additional surgical criteria to THA or TFOI for patients who sought both reconstructions.

#### Data analysis

Demographic data were summarized with descriptive statistics. Kolmogorov-Smirnov testing identified continuous data satisfied normality; therefore, means were compared using paired 2-tailed Student’s *t*-test. Frequencies were compared using Fisher’s exact test. Statistical analysis was performed using Google Sheets (Google LLC, Mountain View, CA). Significance was set at *P* ≤ .05.

#### Data collection

Patients considering osseointegration are asked to complete preoperative QOL surveys (Questionnaire for Persons with a Transfemoral Amputation [QTFA] and Short Form 36 [SF36]) and perform basic mobility tests (timed up and go [TUG] and 6-Minute Walk Test [6MWT]). Patient K-level was determined by the surgeons during clinical examination. Patients are invited to complete the same questionnaires and mobility tests annually. Osseointegration is not withheld from patients who decline to participate in formal research metrics. All osseointegrated patients are enrolled in the registry to optimize follow-up efforts.

Adverse events for all patients were recorded. These included postoperative systemic complications (cardiovascular, circulatory, or pulmonary events), death, or any additional surgical intervention to address issues such as infection or residual limb refashioning for skin redundancy.

#### Implant design, technique, and rehabilitation description

Osseointegration implant principles are summarized as follows. Our practice began osseointegration procedures using the Integrated Limb Prosthetic (Orthodynamics, Lubeck, Germany) but has transitioned over time to using the Osseointegrated Prosthetic Limb (OPL; Permedica Medical Manufacturing, Lecco, Italy). These systems are designed for press-fit fixation, and both consist of 2 components implanted intraoperatively: an intramedullary stem designed to achieve skeletal integration and a dual cone adapter that inserts into the intramedullary component and is passed through a permanent skin portal to create an interface with the attached external prosthetic limb. Our current preference is for osseointegration and transcutaneous externalization in a single surgical event. The surgical technique is similar to this video [10]. Regarding THA associated with TFOI, the 2 reconstructions can be performed simultaneously, or metachronously with either THA or TFOI first, depending on the patient’s rehabilitation priorities. The THA femoral stem has a Morse taper that mates with the TFOI proximal stem’s matched Morse taper, leading to a fully mechanically linked THA + TFOI construct. Accordingly, there is no risk of fracture between the THA and TFOI, and therefore, no plating or other intercalary fixation is utilized. After surgery, patients proceed through postoperative care and the established rehabilitation protocol, which primarily involves (1) progressively increasing static axial loading directly on the prosthesis abutment in the days following TFOI; (2) after 50% weight loading has been achieved (usually 2-3 weeks), increasing axial loading using a temporary lightweight prosthesis; and (3) full weight-bearing axial loading with a personalized prosthesis at 4-6 weeks postoperatively (independent ambulation without walking aides begins at 12 weeks). No casts or splints are used. Follow-up evaluations in our office are scheduled at 3 weeks, 6 weeks, 3 months, 6 months, and annually, or as needed thereafter.

When a THA is performed, there are no additional weight-bearing or activity limitations in addition to that of TFOI (if performed simultaneously). If the THA is performed separately from an acute TFOI, patients are encouraged to mobilize the same or next day, similar to a standard short-stay hip arthroplasty protocol.

## Results

**Figure 2:**
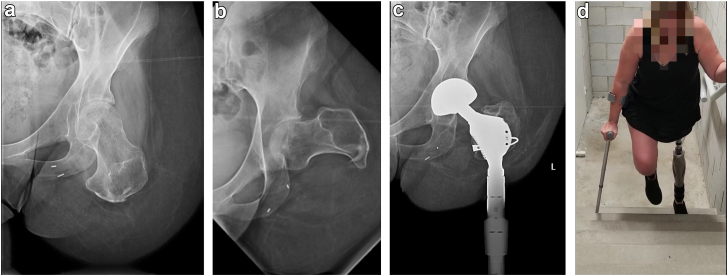
Patient 3 presented as a 35-year-old woman with a history of left transfemoral amputation and also right transtibial amputation performed to manage traumatic injury 10 years prior. She had right transtibial osseointegration (not detailed in this article) and also left THA + TFOI reconstructions. (a, b) Anterior-posterior and lateral radiographs of the left residual femur identify the only amount of bone remaining was to the lesser trochanter. This was too short to wear a prosthesis, rendering her wheelchair-bound. (c) Left hip anterior-posterior radiograph 5 years after simultaneous THA + TFOI identifies the reconstruction; note the femoral component of the THA has a male Morse taper that mates with the female Morse taper of the TFOI implant. (d) A still frame exported from Video 1 shows she walks with the aid of 2 forearm crutches but is able to navigate level surfaces and also stairs; an improvement vs being wheelchair-bound prior to osseointegration.

**Figure 3:**
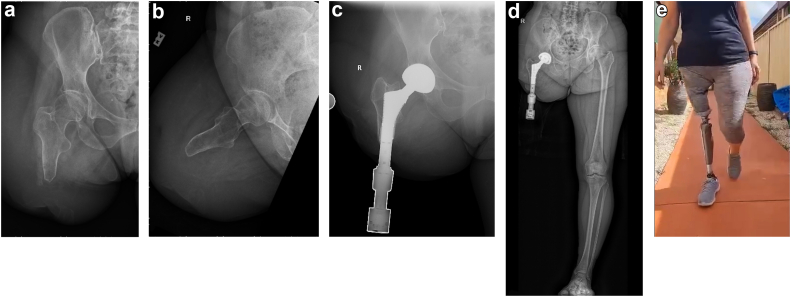
Patient 4 presented as a 53-year-old woman with a proximal right femur amputation performed 41 years prior to manage osteosarcoma. (a, b) Anterior-posterior and lateral radiographs of the right femur identify a residual limb too short to wear a prosthesis, rendering her wheelchair-bound. (c, d) Anterior-posterior right hip and long-standing radiograph taken 5 years after simultaneous THA + TFOI identify the reconstruction; note the femoral component of the THA has a male Morse taper that mates with the female Morse taper of the TFOI implant. (e) A still frame exported from Video 2 taken 6 years after THA + TFOI demonstrates she can walk comfortably in normal tandem gait.

Table 1 presents the patient demographics and also the primary outcome of adverse events and complications prompting additional surgery. There were 8 patients (4 male), all with unilateral THA + TFOI. The average age was 52.8 ± 14.8 years (range 32-73). Seven patients had primary transfemoral amputation performed prior to presentation, at a mean of 20.6 ± 15.5 (range 10-45) years prior to osseointegration (patient 8 had simultaneous amputation with THA + TFOI). Amputation etiology was trauma (three, 38%), cancer (three), infected total knee arthroplasty (one, 13%), and one persistent infection after deformity correction surgery. Three patients (38%) had an osseointegration implant of Integrated limb prosthesis, and 5 (63%) had OPL. Six patients (75%) had simultaneous THA + TFOI, one had TFOI then later THA, and one had THA then later TFOI. Patient 8 died 11 months following THA + TFOI from subsequently diagnosed (unrelated) metastatic pancreatic cancer. Three patients required subsequent surgeries. Two were soft-tissue refashioning (patients 3 and 4). Patient 3 is depicted in Figure 2 and Video 1. Patient 4 is depicted in Figure 3 and Video 2. The third patient (patient 6) underwent 1 hip debridement and arthroplasty retention, with subsequent removal of the TFOI (still retained THA), and returned to the use of crutches with a socket prosthesis. No perioperative complications occurred, no periprosthetic fractures occurred, no joints were explanted, and no further proximal amputations were performed.

**Table 1:** Demographics and primary outcomes.

Patient	Age/Sex/Side	Amputation etiology	Implant type	THR/OI Stages	Additional surgery or complications	Years from amputation to osseointegration	Follow-up years
1	43/F/L	Trauma	ILP	TFOI then THR	-	16.7	9.9
2	64/M/R	Osteosarcoma	ILP	Concurrent	-	17.9	9.6
3	35/F/L	Trauma	OPL	Concurrent	Refashioning ×3	10.5	8.7
4	53/F/R	Osteosarcoma	ILP	Concurrent	Refashioning ×2. Methicillin resistant staph aureus grown from aspiration 6 y post OI. Oral trimethoprim/sulfamethoxazole began 6 y and 6 mo post OI. Ceased 3 mo later. Has maintained stable clinical suppression in subsequent 6 mo to current.	41.2	7.3
5	53/M/L	Trauma	OPL	THR then TFOI	-	10.5	6.5
6	32/F/R	Osteosarcoma	OPL	Concurrent	Hip debridement after 5 y 3 mo, persistent *Finegoldia magna* infection of TFOI led to TFOI removal after 5 y 7 mo with THR retention.	23.0	6.0
7	65/M/R	Ischemia/gangrene following HTO	OPL	Concurrent	-	45.1	2.9
8	73/M/L	Infection following TKR	OPL	Concurrent	Deceased (unrelated new cancer)	0	0.9
Mean or Total	52.8 ± 14.8; 4/8 = 50% male; 4/8 = 50% left		3/8 = 38% ILP; 5/8 = 63% OPL			20.6 ± 15.5	6.5 ± 3.2

ILP, integrated limb prosthesis; OI, osseointegration; OPL, osseointegrated prosthetic limb; TFOI, transfemoral osseointegration; THR, total hip replacement; HTO, high tibia osteotomy.

**Figure 4:**
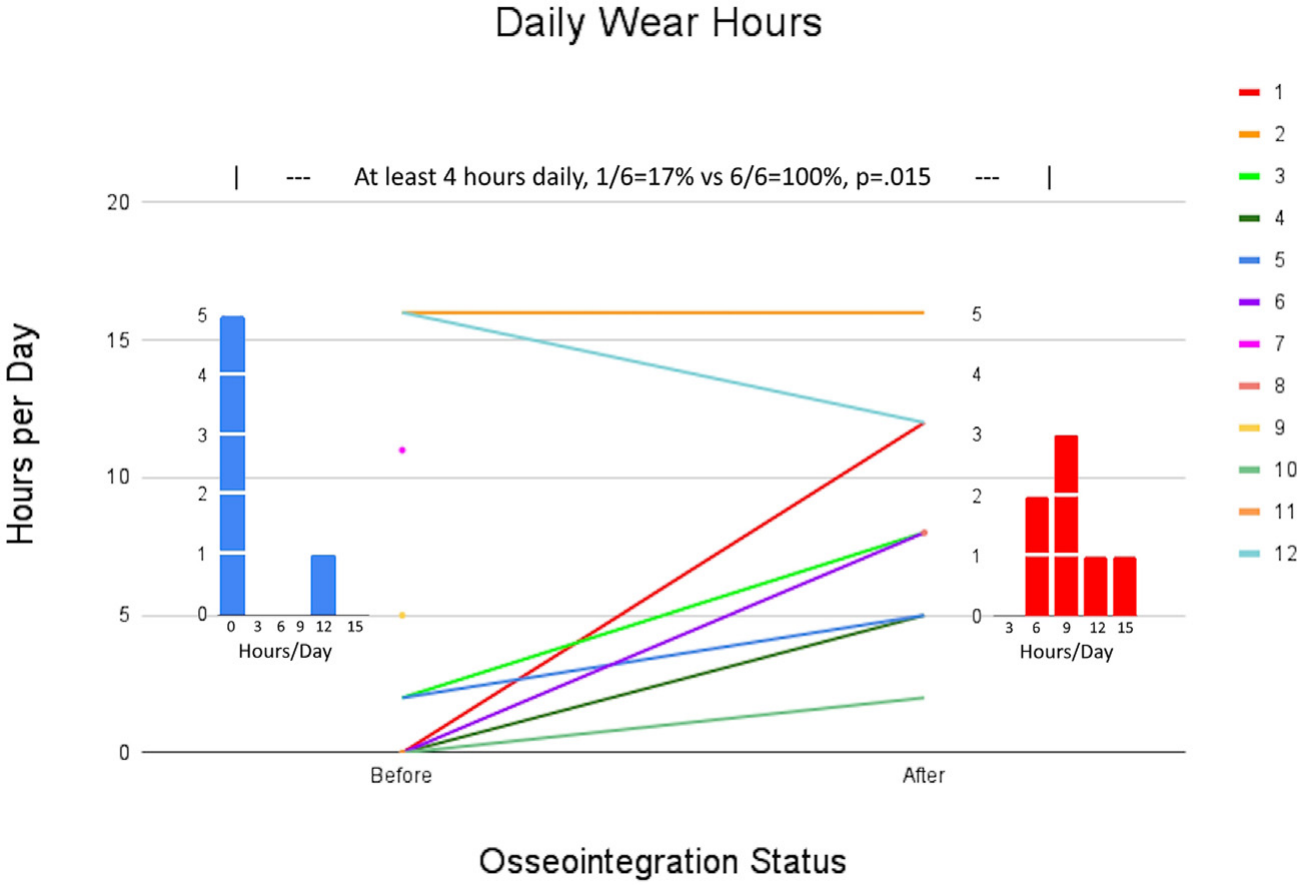
Daily prosthesis wear time. No patients decreased prosthesis wear time from before vs after osseointegration. The proportion of patients who wore their prosthetic limb at least 4 hours daily increased from 1/6 = 17% to 6/6 = 100% (*P* = .015).

**Figure 5:**
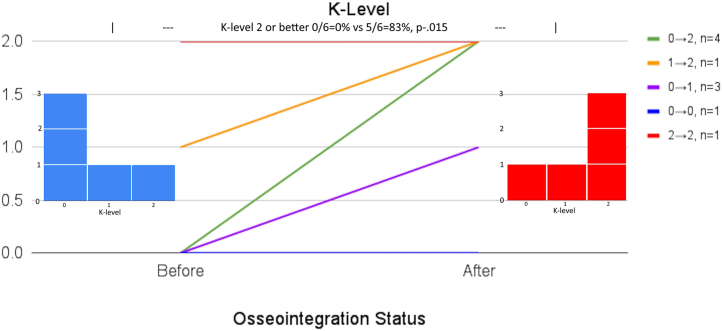
K-level of patients. All patients increased their K-level, and none decreased. The proportion of patients who were at least K-level 2 increased from 0/6 = 0% before vs 5/6 = 83% after (*P* = .015).

Table 2 presents 1 secondary outcome: mobility data. Preoperative and postoperative daily wear hours, K-level, 6MWT, and TUG are compared. Patient 8’s mobility and QOL data were excluded because he died prior to 2 years following surgery, which was the minimum time frame of interest for this study. All patients maintained or improved prosthesis wear hours; no patient reduced daily wear hours. Of 6 patients who responded both before and after osseointegration, one (17%) wore their prosthesis at least 4 hours daily before vs all (100%) who did afterward (*P* = .015; Fig. 4). K-levels improved in all 6 responding patients. Zero patients (0%) were at least K2 before vs 5/6 = 83% after (*P* = .015; Fig. 5). The most remarkable finding is that all amputees who were wheelchair-bound (K0) preoperatively achieved ambulation following THA + TFOI, with 4/5 = 80% K2. Patients 1, 3, 4, and 6 completed both preoperative and postoperative 6MWT, which improved from 0 ± 0 m to 285.3 ± 31.3 m (*P* < .001).

**Table 2:** Mobility data.

Patient	Daily wear hours		K-level		6MWT		TUG	
	Before	After	Before	After	Before	After	Before	After
1	0	13-15	0	2	WB	325	WB	9.54
2	16	16	1	2	N/A	277.5	13.6	12.2
3	1-3	7-9	0	2	WB	240	WB	15
4	0	4-6	0	2	WB	284	WB	12
5	1-3	4-6	0	1	WB	N/A	WB	N/A
6	0	7-9	0	2	WB	300	WB	9.2
7	10-12	N/A	1	N/A	100	N/A	14	N/A
8	Full leg	Died	Full leg	Died	WB	Died	WB	Died

Patient 8’s data are presented in strikeout format because he was excluded from mobility evaluation, since he died prior to the minimum follow-up time for this study’s inclusion criteria. It is presented in the table for transparency purposes.

Table 3 evaluates the other secondary outcome: QOL data in the form of QTFA and SF-36 surveys. Improvements (although not achieving statistical significance) were recorded in mean QTFA Global (39.6 ± 24.9 vs 64.6 ± 4.2, *P* = .134), mobility (40.7 ± 27.1 vs 66.2 ± 26.7, *P* = .179), and problem (52.6 ± 36.6 vs 13.8 ± 8.3, *P* = .107) scores. Three out of 4 patients with complete survey data exhibited improvements or remained the same in global and mobility scores. Insignificant changes were also recorded in SF36 Mental (56.0 ± 11.3 vs 53.6 ± 3.8, *P* = .659) and Physical (31.4 ± 11.5 vs 39.4 ± 8.6, *P* = .348) scores.

**Table 3:** Quality of life survey data.

Patient	QTFA global		QTFA mobility		QTFA problem		SF-36 mental		SF-36 physical	
	Before	After	Before	After	Before	After	Before	After	Before	After
1	N/A	91.67	22.22	96.67	88.33	0	46.65	57.82	21.32	52.51
2	66.67	66.67	73.89	75	25	13.75	59.86	52.27	37.87	39.6
3	8.33	66.67	11.11	27.78	77.9	27.78	40.05	49.56	32.62	29.32
4	50	66.67	63.89	52.22	3.33	20.42	72.24	49.16	19.05	42.8
5	33.33	58.33	32.22	79.44	68.33	20	58.398	57.48	27.637	41.46
6	N/A	50	N/A	62.22	N/A	31.67	58.6	54.84	50.06	30.39
7	16.67	N/A	61.11	N/A	43.75	N/A	29	N/A	30.55	N/A
Mean ± SD	39.6 ± 24.9	64.6 ± 4.2	40.7 ± 27.1	66.2 ± 26.7	52.6 ± 36.6	13.8 ± 8.3	56.0 ± 11.3	53.6 ± 3.8	31.4 ± 11.5	39.4 ± 8.6
*P*-value	*P* = .134		*P* = .179		*P* = .107		*P* = .659		*P* = .348	

Patient 8 was excluded from QOL survey evaluation because he died prior to the minimum follow-up time for this study’s inclusion criteria.

## Discussion

This report expands upon a prior pilot study [9] and was undertaken to describe the medium-term (minimum 2 year) experience of patients who underwent THA + TFOI. The most important finding is that the presence of a permanent transcutaneous skin portal for the osseointegrated prosthesis anchor does not appear to pose an inevitable risk for infection of the prosthetic THA. Only one patient (13%) developed a prosthetic joint infection, which was managed by removal of the TFOI component, with debridement and retention of the THA components. Several other findings are notable. All patients who presented confined to wheelchair locomotion achieved and have retained independent ambulation. No patients had loss of bone-implant fixation. No patient had adverse events prompting more proximal amputation, such as hip disarticulation.

Arthritic hip pain reduces QOL similar to chronic cardiac disease [11]. THA consistently reduces pain and improves mobility [12]. As with any surgical procedure, there are potential related complications, such as infection [13], periprosthetic fracture [14], and dislocation [15]. Despite these potentially devastating complications [16,17], THA is considered among the most monumentally important reconstructive surgeries [18]. This results from continued exceptional innovation and improvement to implant designs, surgical techniques, protocols, patient selection, and preoperative optimization. Still, several complications remain onerous, particularly in complex scenarios. A 2023 systematic review of hip fusion conversion to arthroplasty reported the following complication profile: heterotopic ossification (14%), aseptic loosening (5%), intraoperative fracture (4%), greater trochanteric nonunion (3%), deep infection (3%), neural injuries (3%), and dislocation (2%) [19]. An exceptionally important point is that current total joint infection criteria consider a direct sinus to the joint to be a major criterion for infection [20]. If that standard were to be applied to this cohort, the transcutaneous portal through which the osseointegration implant passes could be considered a sinus, and the THA considered infected by definition. However, the clinical reality is that signs and symptoms of prosthetic joint infection only occurred in the single patient. Two years ought to have been sufficient time for an actual sinus to result in such an infection for the predominance of patients. We believe it is important to specifically emphasize that this has not happened, as this question was a major motivation underlying this study. We do not have a full explanation for why infection does not occur despite the skin portal. The following statement is conjecture, not directly investigated data. It may be that a substantial (not necessarily entirely singular) barrier to infection for osseointegration might be that bone growing onto (or, in actuality, within 1 μm of [21]) the implant surface creates a barrier against bacterial ingress. This “race to the implant” theory was likely first popularized by Gristina [22].

Lower-extremity amputation, specifically transfemoral amputation, imposes substantial mobility and QOL challenges [23,24]. The skin-socket interface to the prosthetic leg likely contributes greatly to those challenges [1,2]. Osseointegrated prosthesis anchors usually achieve improved mobility and enhanced QOL [7,8] by eliminating the socket. The better implant-limb stability achieves reduced metabolic demand [25] and improves balance [26,27]. Nevertheless, there are complications associated with TFOI. The need for removal to address infection or implant loosening appears to be under 5% [28]. Hip fracture from falls or similar trauma has been reported at 6%; following fracture care, patients regain mobility better than they had in their socket prosthesis [[29], [30], [31]]. Increasing evidence supports the safety and benefit of osseointegration for an expanding range of patients with lower-extremity issues, notably complex regional pain syndrome [32], peripheral vascular disease [33], infection after total knee arthroplasty [34], burns trauma [35], very short residual limbs [36,37], irradiated limbs [38], and patients with hip disarticulation [39]. Patients with hip arthritis and transfemoral amputation are another important cohort to consider.

Many of the patients reported in this study had very short residual femurs, rendering them unable to use a transfemoral prosthesis. Such patients may function essentially as a hip disarticulation patient, requiring substantial additional metabolic demand for ambulation [40]. Often these patients’ mobility and QOL are remarkably poor [41]. More than half may give up attempting prosthetic rehabilitation [42]. While osseointegration can also be performed for patients with a hip disarticulation [39], preserving a functional joint presumably should confer better performance. THA + TFOI appears able to retain the use of even exceptionally short residual femurs, provided the surrounding muscles are functional. Conceivably, in a “worst case scenario,” a patient may end up with complications prompting formal hip disarticulation, rendering them equal to what they would have been without THA + TFOI.

Combining the rehabilitation benefit of THA and TFOI is desirable for patients struggling with limb loss and painful hip joint arthritis. Both THA and TFOI have been proven to be safe and effective for the vast majority of patients. Severe pain, with associated immobility, results in a devastating reduction in QOL. The limitations of socket rehabilitation are sometimes even associated with suicide [43]. In that context, it is reasonable to offer therapeutic interventions to such patients who express severe dissatisfaction with their mobility and QOL, so long as patients comprehend the potential risks: This is the patient’s right to beneficence [44]. TFOI has less than 1% risk of associated mortality or more proximal limb amputation [6]. This knowledge helps patients self-assess whether the likelihood for improving their mobility, reducing pain and other problems associated with TSP-aided mobility, and/or alleviating arthritic joint pain in an amputated limb is consistent with their individual sense of benefit and risk. Analyzing the outcomes improves the shared decision-making for future patients. While THA + TFOI likely has many aspects that would benefit from technique or design improvements, most importantly to reduce the risk of infection through the implant-skin portal, in our opinion, the current risk-to-benefit profile is reasonable to continue offering this rehabilitation opportunity to well-informed and appropriately selected patients.

This study is the first to evaluate the mid-term (minimum 2 years) outcomes of THA + TFOI, and there are important limitations to consider: primarily, the small sample size of eight patients and the retrospective design’s susceptibility to selection, performance, and recall bias. These biases may influence the results in ways that are difficult to predict. Similarly, by definition of the length of the study, long-term outcomes remain uncertain, particularly regarding infection. An additional important limitation is that the procedures were performed in a practice with high volume of both hip arthroplasty and also osseointegration; the technical outcomes may not necessarily be generalizable. We believe a strength of this study is the maintained proof of feasibility of this reconstructive rehabilitation surgery, and in our opinion, improvement of mobility and QOL. Although some patients had adverse events, the majority demonstrated measurable improvements. It is emphasized that this study was not designed nor intended to be a definitive examination of the appropriateness of this rehabilitative reconstruction or to establish expected outcomes of THA + TFOI. Rather, it is intended to demonstrate the feasibility and general safety of the current techniques and technology, to provide awareness about this intervention for interested clinicians and patients, and hopefully to provide information for additional improvements to implants and techniques. As those are better defined, more consistent improvements may be provided with fewer adverse events for patients who may later benefit from this reconstructive option.

## Conclusions

THA + TFOI appears to be a reasonable rehabilitation option for transfemoral amputees who have arthritic hip pain and are dissatisfied with their rehabilitation situation using a TSP. Although infection can occur, the presence of a permanent transcutaneous skin portal for the osseointegrated prosthesis anchor does not appear to pose an inevitable risk of prosthetic hip infection. Further judicious use with appropriately selected and counseled patients appears warranted.

## Conflicts of interest

Munjed Al Muderis is the sole beneficiary of Osseointegration Holdings Pty Ltd (“OH”) and Osseointegration International Pty Ltd (“OI”). OI exclusively distributes the OPL implant system worldwide. OH owns the rights and patents to the OPL implant system. Other authors have no competing financial interest.

For full disclosure statements refer to https://doi.org/10.1016/j.artd.2024.101463.

## CRediT authorship contribution statement

**Jason Shih Hoellwarth:** Writing – review & editing, Writing – original draft, Project administration, Investigation, Formal analysis, Data curation, Conceptualization. **Amanullah Haidary:** Writing – review & editing, Writing – original draft, Data curation. **Kevin Tetsworth:** Writing – review & editing, Writing – original draft, Formal analysis, Conceptualization. **Atiya Oomatia:** Writing – review & editing, Writing – original draft, Formal analysis, Data curation. **Munjed Al Muderis:** Writing – review & editing, Writing – original draft, Investigation, Data curation, Conceptualization.

## Appendix A Supplementary data

Conflict of Interest Statement for Al Muderis

Conflict of Interest Statement for Haidary

Conflict of Interest Statement for Hoellwarth

Conflict of Interest Statement for Oomatia

Conflict of Interest Statement for Tetsworth
