# Supplementary material for: Transfemoral Osseointegration in Association With Total Hip Replacement: Observational Cohort Study of Patients With Follow-Up Exceeding 2 Years
Source: Arthroplast Today. 2024 Jul 20;28:101463. doi: 10.1016/j.artd.2024.101463 (PMC11295706; doi:10.1016/j.artd.2024.101463)
Supplement: Conflict of Interest Statement for Al Muderis [file mmc1.pdf]

INDIVIDUAL CONFLICT OF INTEREST STATEMENT

American Association of Hip and Knee Surgeons

(Adopted from the American Academy of Orthopaedic Surgeons disclosure statement)

The following form **must be filled out completely and submitted by each author (example, 6 authors, 6 forms). All items require a response. If there is no relevant disclosure for a given item, enter "*None.*"**

Manuscript Title

1. Royalties from a company or supplier (The following conflicts were disclosed)

Munjed Al Muderis is the sole beneficiary of Osseointegration Holdings Pty Ltd ("OH") and Osseointegration International Pty Ltd ("OI"). OI exclusively distributes the OPL implant system worldwide. OH owns the rights and patents to the OPL implant system.

2. Speakers bureau/paid presentations for a company or supplier (The following conflicts were disclosed)
None

3A. Paid employee for a company or supplier (The following conflicts were disclosed)

none

3B. Paid consultant for a company or supplier (The following conflicts were disclosed)
Munjed Al Muderis is the sole beneficiary of Osseointegration Holdings Pty Ltd ("OH") and Osseointegration International Pty Ltd ("OI"). OI exclusively distributes the OPL implant system worldwide. OH owns the rights and patents to the OPL implant system.

3C. Unpaid consultants for a company or supplier (The following conflicts were disclosed)
Munjed Al Muderis is the sole beneficiary of Osseointegration Holdings Pty Ltd ("OH") and Osseointegration International Pty Ltd ("OI"). OI exclusively distributes the OPL implant system worldwide. OH owns the rights and patents to the OPL implant system.

4. Stock or stock options in a company or supplier (The following conflicts were disclosed)
Munjed Al Muderis is the sole beneficiary of Osseointegration Holdings Pty Ltd ("OH") and Osseointegration International Pty Ltd ("OI"). OI exclusively distributes the OPL implant system worldwide. OH owns the rights and patents to the OPL implant system.

5. Research support from a company or supplier as a Principal Investigator (The following conflicts were disclosed)
none

6. Other financial or material support from a company or supplier (The following conflicts were disclosed)
Munjed Al Muderis is the sole beneficiary of Osseointegration Holdings Pty Ltd ("OH") and Osseointegration International Pty Ltd ("OI"). OI exclusively distributes the OPL implant system worldwide. OH owns the rights and patents to the OPL implant system.

7. Royalties, financial or material support from publishers (The following conflicts were disclosed)
Munjed Al Muderis is the sole beneficiary of Osseointegration Holdings Pty Ltd ("OH") and Osseointegration International Pty Ltd ("OI"). OI exclusively distributes the OPL implant system worldwide. OH owns the rights and patents to the OPL implant system.

8. Medical/Orthopaedic publications editorial/governing board (The following conflicts were disclosed)
none

9. Board member/committee appointments for a society (The following conflicts were disclosed)
None

Each author must sign AND print or type his/her name, date and submit a separate form

In addition, one BLINDED Conflict of Interest form (no author names used) should be submitted per manuscript with all author disclosures.

Munjed Al Muderis	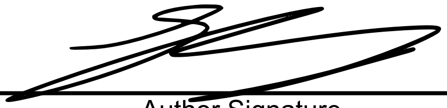	11/11/2023
Author Name (Print or Type)	Author Signature	Date
